# Post-Acute Nasal Expression of ACE2, TMPRSS2, FURIN, and NRP1 in Relation to COVID-19 Severity: A Multicenter Cross-Sectional Study

**DOI:** 10.3390/diagnostics16182896

**Published:** 2026-09-09

**Authors:** Ana María Piqueras-Sánchez, José Francisco López-Gil, Diego Hellín-Meseguer, Juan Cabezas-Herrera, Ginés Francisco Blesa-Llaona, José Meseguer-Cabezas, Enrique Bernal-Morell, Alfredo Minguela-Puras, Francisco Mateo Piqueras-Pérez, José Antonio Díaz-Manzano

**Affiliations:** 1 Department of Otolaryngology, Virgen de la Arrixaca University Clinical Hospital (HCUVA), 30120 Murcia, Spain; diego.hellin@um.es (D.H.-M.); gblesa@gmail.com (G.F.B.-L.); pepechinatto@gmail.com (J.M.-C.); jdiazmanzano@hotmail.com (J.A.D.-M.); 2 Department of Ophthalmology, Optometry, Otorhinolaryngology, and Pathological Anatomy, University of Murcia (UMU), 30100 Murcia, Spain; 3 School of Medicine, Universidad Espíritu Santo, Samborondón 092301, Ecuador; 4 Faculty of Health Sciences, Universidad Autónoma de Chile, Temuco 4810101, Chile; 5 Biomedical Research Institute of Murcia (IMIB), 30120 Murcia, Spain; jcabezas@um.es (J.C.-H.); ebm.hgurs@gmail.com (E.B.-M.); alfredo.minguela@carm.es (A.M.-P.); 6 Molecular Therapy and Biomarkers Research Group, Virgen de la Arrixaca University Clinical Hospital (HCUVA), 30120 Murcia, Spain; 7 Department of Internal Medicine, Section of Infectious Diseases, Reina Sofía Universitary Hospital (HRSU), 30003 Murcia, Spain; 8 Immunology Service, Virgen de la Arrixaca University Clinical Hospital (HCUVA), 30120 Murcia, Spain; 9 Department of Otolaryngology, Morales Meseguer Hospital, 30008 Murcia, Spain; fmpiquerasp@yahoo.es

**Keywords:** COVID-19, SARS-CoV-2, ACE2, TMPRSS2, FURIN, NRP1, nasal epithelium, post-acute phase, disease severity

## Abstract

**Background/Objectives**: Host factors angiotensin-converting enzyme 2 (ACE2), transmembrane protease-serine 2 (TMPRSS2), furin paired basic amino acid cleaving enzyme (FURIN), and neuropilin-1 (NRP1) facilitate severe acute respiratory syndrome coronavirus 2 (SARS-CoV-2) entry, but it is unclear whether their upper-airway expression after recovery is associated with the severity of the preceding acute illness. We examined the association between post-acute expression of these genes and previous coronavirus disease 2019 (COVID-19) severity. **Methods**: This multicenter cross-sectional study included 104 adults with polymerase chain reaction (PCR)-confirmed COVID-19 during the first two pandemic waves in Murcia, Spain. Nasal and/or oropharyngeal swabs were collected in the post-acute phase, at a median of 75 days after symptom onset, and transcript levels were quantified by quantitative real-time PCR. Severity was categorized using the World Health Organization (WHO) Clinical Progression Scale, and associations were evaluated using logistic regression adjusted for age, sex, and race/ethnicity. **Results**: In the primary analyses using continuous expression measures, none of the four genes was significantly associated with severity. In exploratory tertile-based analyses, the intermediate ACE2 tertile (odds ratio [OR] = 0.17, 95% confidence interval [CI] 0.05–0.61; *p* = 0.007) and intermediate NRP1 tertile (OR = 0.29, 95% CI 0.10–0.88; *p* = 0.030) were associated with lower odds of severe disease; no significant associations were observed for the high tertiles or for FURIN or TMPRSS2. **Conclusions**: Primary adjusted analyses did not reveal statistically significant associations between post-acute nasal expression of ACE2, TMPRSS2, FURIN, or NRP1 and COVID-19 severity. Because expression was measured after clinical recovery, these associations cannot be interpreted as predictors of acute severity and may instead reflect persistent molecular remodeling after more severe disease; reverse causation cannot be excluded. Longitudinal studies with acute-phase and serial post-acute sampling and healthy controls are needed.

## 1. Introduction

Coronavirus Disease 2019 (COVID-19) emerged as a major global health emergency, with the first pandemic waves generating unprecedented mortality, morbidity, and a profound sociohealth crisis [1,2]. These circumstances underscored the critical necessity of advancing research on this pathogen and of establishing effective prevention and treatment strategies. Severe acute respiratory syndrome coronavirus 2 (SARS-CoV-2), the causative agent of COVID-19, primarily gains cellular entry through angiotensin-converting enzyme 2 (ACE2) [3,4]. ACE2 is a membrane-bound enzyme involved in the regulation of the renin–angiotensin–aldosterone system, which governs blood pressure and fluid homeostasis [5]. Its tissue expression is heterogeneous across individuals and modulated by demographic and clinical variables such as sex, age, race, and comorbid conditions [4,5,6,7,8,9].

Beyond ACE2, several other host proteins are indispensable for SARS-CoV-2 infection, including transmembrane protease-serine 2 (TMPRSS2), furin paired basic amino acid cleaving enzyme (FURIN), and neuropilin-1 (NRP1). TMPRSS2 enables viral internalization by priming the spike protein [10], while FURIN contributes to viral protein maturation and promotes spike-ACE2 binding [11,12]. NRP1 has been implicated in olfactory dysfunction, a hallmark symptom of COVID-19, possibly by facilitating viral neurotropism [13,14]. Mechanistically, SARS-CoV-2 cell entry depends on spike protein engagement with ACE2 as the principal receptor; TMPRSS2-mediated and FURIN-mediated cleavage events activate this process, and NRP1 may act as a co-receptor to amplify infectivity [2,15].

This study examined post-acute upper-airway mRNA expression of ACE2, TMPRSS2, FURIN, and NRP1 in individuals from the Region of Murcia who had recovered from COVID-19, with the goal of determining whether the post-acute nasal expression of these SARS-CoV-2 entry factors was linked to the clinical severity experienced during the acute illness.

## 2. Materials and Methods

### 2.1. Study Design and Population

This multicenter cross-sectional study combined retrospective clinical data with a single upper-airway biological sample collected during the post-acute period. The objective was to evaluate whether post-acute mRNA expression of four SARS-CoV-2 entry-related host factors (ACE2, TMPRSS2, FURIN, and NRP1) was associated with the severity of the preceding acute COVID-19 illness in adults recruited during the first and second pandemic waves.

Biological sampling was performed once during post-acute recovery, at a median of 75 days after symptom onset, when all participants were clinically stable and free of active disease. Clinical follow-up continued for up to 120 days from symptom onset. Sampling after recovery was intended to reduce the immediate effects of viral replication, acute inflammation, and transient epithelial injury on expression measurements. However, because pre-infection and acute-phase samples were unavailable, this design cannot characterize expression during acute infection or establish temporal direction; post-acute values may reflect persistent consequences of the preceding illness.

Of 201 adults initially identified with polymerase chain reaction (PCR)-confirmed COVID-19, 104 were enrolled; the remainder declined participation or were excluded because of technical difficulties in sample collection or processing. The analytical cohort comprised 69 men (66.3%) and 35 women (33.7%), with a median age of 58.5 years (interquartile range [IQR] 16.2); most participants self-identified as Caucasian (81.7%, *n* = 85). The final sample size may have limited statistical power to detect small or moderate associations, and the estimates should therefore be interpreted cautiously.

An adapted version of the WHO Clinical Progression Scale as summarized in Table 1 [16] was used to classify disease severity. For descriptive purposes, WHO groups 2 (hospitalization with ambient oxygen) and 3 (low-flow oxygen therapy) were combined into a single "hospitalization with oxygen" category, given the limited number of participants in each individual group. For regression analyses, categories were consolidated to retain an adequate number of participants in each outcome group and improve estimate stability given the available sample size.

**Table 1:** World Health Organization severity scale.

Group	Clinical Criteria
1	No hospitalization
2	Hospitalization with ambient oxygen
3	Low-flow oxygen therapy
4	Non-invasive ventilation or high-flow oxygen
5	Invasive ventilation
6	Death

### 2.2. Ethical Approval

All procedures adhered to the ethical standards established by the Declaration of Helsinki. Institutional review board approval was granted by the Ethics Committee of the Virgen de la Arrixaca University Clinical Hospital (Internal Code: 2020-10-8-HCUVA), with jurisdiction extended to all collaborating centers. Each participant gave written informed consent before enrollment.

### 2.3. Procedures

Samples were obtained by nasal and/or oropharyngeal swabbing. Upon arrival at the laboratory, each swab was agitated in an RNA-preserving solution and centrifuged at 2500× *g* for 5 min using a Beckman Allegra X-12R centrifuge. Total RNA was extracted from the resulting epithelial cell pellet using the InviTrap^®^ Spin Universal RNA Mini Kit (Stratec Molecular, Robert-Roessle-Str. 10, Building 8513125, Berlin, Germany; ref. 1060100200), according to the manufacturer’s instructions, and stored at −80 °C until analysis.

Before reverse transcription, RNA samples were heated at 80 °C for 10 min and rapidly cooled on ice to minimize the formation of secondary structures. Complementary DNA (cDNA) was synthesized using the GeneAmp RNA PCR Core Kit (Applied Biosystems/Roche, Applied Biosystems, Foster City, CA, USA; Cat. No. N808-014) and random hexamer primers in a 2720 Thermal Cycler (Applied Biosystems, Foster City, CA, USA). The reverse-transcription conditions were 25 °C for 10 min, 42 °C for 15 min, and 99 °C for 5 min. The resulting cDNA was cooled on ice and stored at −20 °C until amplification.

Primers for ACE2, TMPRSS2, FURIN, NRP1, and the housekeeping gene GAPDH were designed using Primer3 based on RNA sequences available from the National Center for Biotechnology Information. Exact oligonucleotide sequences were not retained in the archived laboratory records available to the authors and therefore cannot be reported; amplification specificity was assessed by melting-curve analysis and by agarose gel electrophoresis and/or sequencing of amplification products.

After an initial activation step at 95 °C for 30 s, amplification was performed for 40 cycles consisting of denaturation at 95 °C for 5 s, primer annealing at 55–60 °C for 10 s, and extension at 72 °C for 10–12 s. Negative controls were included by omitting cDNA, primers, or Taq polymerase and replacing the corresponding volume with water. A post-amplification melting-curve analysis was performed using a temperature gradient of 0.1 °C/s to assess amplification specificity. The identity of the amplification products was additionally evaluated by agarose gel electrophoresis and/or sequencing.

The molecular variables were the normalized mRNA transcript levels of ACE2, TMPRSS2, FURIN, and NRP1 in nasal and/or oropharyngeal mucosal specimens. Expression was normalized to glyceraldehyde-3-phosphate dehydrogenase (GAPDH), which served as the housekeeping reference gene. Quantification was performed using the LightCycler^®^ Software version 4.1 (Roche Diagnostics, Mannheim, Germany) and the Fit Points algorithm. In the absence of a healthy control group, an external calibrator, or pre-infection baseline data, results could not be expressed as fold changes relative to an external reference. Consequently, normalized relative expression units were generated for each participant and used for subsequent comparisons across severity strata. The primers used for ACE2, TMPRSS2, FURIN, NRP1, and GAPDH were designed using Primer3 against the corresponding NCBI reference transcripts. The exact forward and reverse primer sequences used in the RT-qPCR assays are available from the corresponding author upon reasonable request.

Participant-reported sociodemographic information included age, biological sex, and self-identified racial/ethnic background. Clinical covariates available in the study database included smoking status (coded as never vs. ever smoker for regression), hypertension, diabetes mellitus, and the Charlson comorbidity index; these variables were complete for all 104 participants. In the cohort, 26.0% were ever smokers (n = 27), 37.5% had hypertension (n = 39), 21.2% had diabetes mellitus (n = 22), and the median Charlson index was 1 (IQR 2).

For regression analyses, WHO groups 1–3 were combined as non-severe, comprising participants who received no ventilatory support or low-flow oxygen, whereas groups 4–5 were combined as severe, comprising participants who required high-flow oxygen, non-invasive ventilation, or invasive mechanical ventilation; no deaths occurred. This dichotomization was chosen to maintain adequate group outcome sizes and improve model stability in the cohort of 104 participants.

The scale spans a clinical continuum from ambulatory mild/moderate management through inpatient care and, at its most severe end, intensive care unit admission.

### 2.4. Statistical Analysis

Associations between post-acute gene expression and COVID-19 severity were evaluated using robust logistic regression (M-estimation via the *glmrob* function, *robustbase* package in R). Primary and tertile-based models were adjusted for age, sex, and race/ethnicity. Tertile-based models were considered exploratory. Missing data were addressed using multiple imputation by chained equations (MICE; predictive mean matching, 9 imputed datasets, 5 iterations). Models were fitted on a single completed dataset. Sensitivity models additionally adjusted for ever smoking, hypertension, diabetes mellitus, and Charlson comorbidity index using complete cases. Exploratory logistic models also evaluated associations between gene expression and olfactory dysfunction or dysgeusia.

## 3. Results

### 3.1. Demographic and Clinical Characteristics

Table 2 presents the demographic and clinical characteristics of the 104 participants. Median age was 58.5 years (IQR 16.2); men accounted for 66.3% (n = 69), and Caucasian participants represented 81.7% (n = 85) of the cohort.

Based on the adapted WHO Clinical Progression Scale, 61.5% of participants required hospital admission (n = 64), whereas 38.5% were managed as outpatients (n = 40). For the primary outcome, 58.7% were classified as non-severe (n = 61) and 41.3% as severe because they required high-flow oxygen, non-invasive ventilation, or invasive mechanical ventilation (n = 43). Invasive mechanical ventilation was required in 10.6% (n = 11) (Table 2). Among sensory manifestations, gustatory dysfunction (dysgeusia) was documented in 36.5% of participants (n = 38), and, in the imputed database, olfactory dysfunction was reported in 37.5% (n = 39).

Median normalized relative expression levels in the database with missing data were 212.3 units (IQR 105.1) for FURIN, 2098.3 units (IQR 1271.3) for ACE2, 1904.3 units (IQR 1627.6) for NRP1, and 113.0 units (IQR 54.4) for TMPRSS2.

### 3.2. Continuous-Expression Models

Continuous post-acute expression measures were evaluated in unadjusted and adjusted binary logistic regression models (Table 3).

No continuous-expression predictor reached statistical significance. In adjusted models, the ORs per 100-unit increment were 1.38 for FURIN (95% CI 0.88–2.17; *p* = 0.157), 1.00 for ACE2 (95% CI 0.96–1.05; *p* = 0.890), 1.00 for NRP1 (95% CI 0.98–1.02; *p* = 0.788), and 0.93 for TMPRSS2 (95% CI 0.57–1.51; *p* = 0.764). Accordingly, these data did not support a trend or statistically significant association for FURIN or the other genes (Table 3).

### 3.3. Tertile-Based Exploratory Models

Expression was then categorized into low, intermediate, and high tertiles to explore potential non-linear associations with previous COVID-19 severity (Table 4).

**Figure 1:**
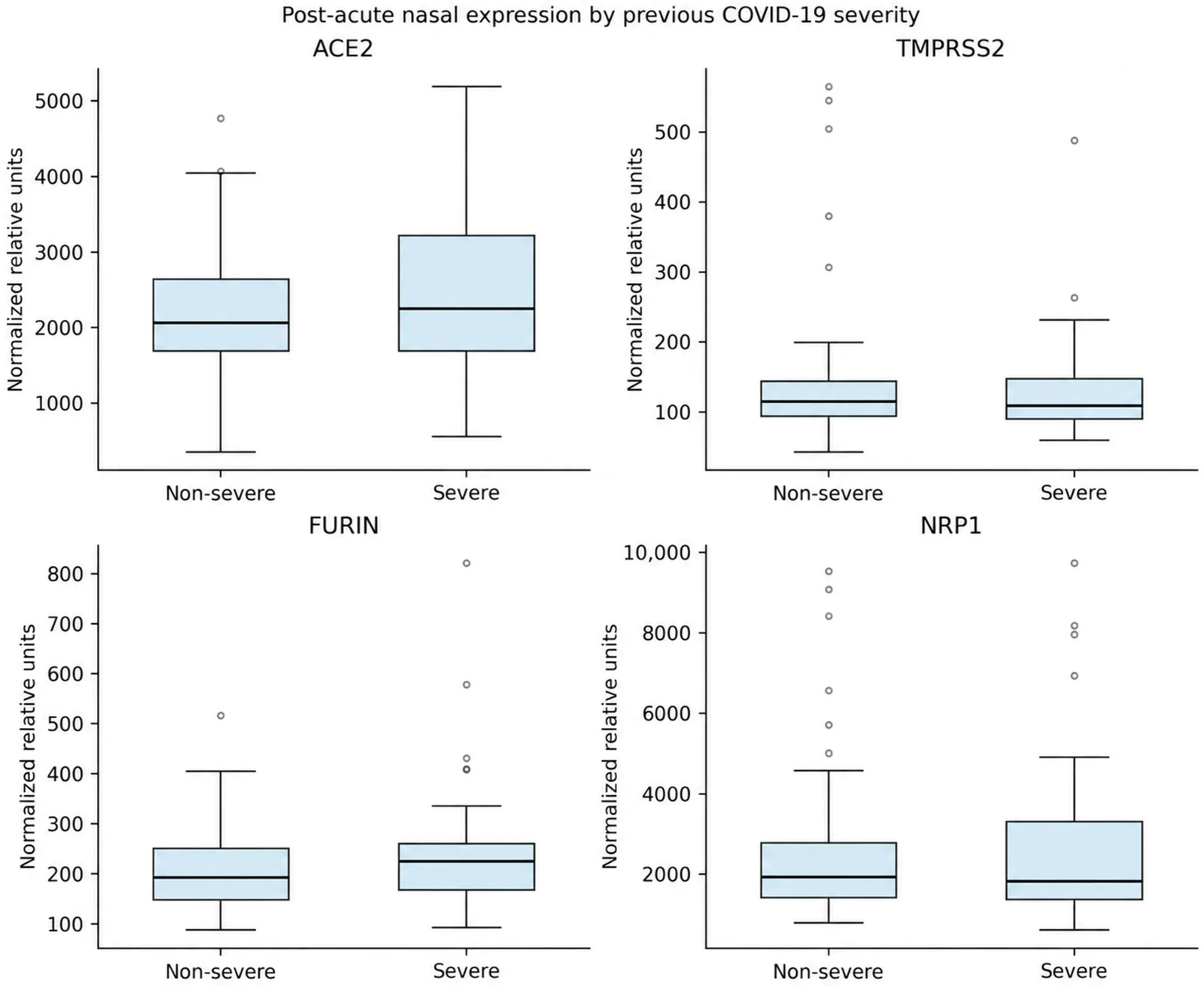
Post-acute nasal expression of ACE2, TMPRSS2, FURIN, and NRP1 by previous COVID-19 severity.

Continuous-expression results are reported in Section 3.2 and Table 3; the present section focuses on exploratory tertile models (Table 4). Distributions of post-acute expression by previous severity are shown in Figure 1.

Exploratory tertile analyses revealed that the intermediate ACE2 tertile was independently associated with substantially reduced odds of severe illness following adjustment for age, sex, and race/ethnicity (OR = 0.17, 95% CI 0.05–0.61; *p* = 0.007), and a comparable protective pattern was seen for the intermediate NRP1 tertile (OR = 0.29, 95% CI 0.10–0.88; *p* = 0.030) (Table 4). High expression levels of either receptor were not significantly associated with severity. No tertile-level associations reached significance for FURIN or TMPRSS2 (Table 4). Figure 1 displays the distribution of normalized relative expression for each gene by previous severity stratum.

### 3.4. Sensitivity Analyses Adjusting for Smoking and Comorbidities

Because smoking status, hypertension, diabetes mellitus, and Charlson comorbidity index were available for the cohort, we repeated the adjusted logistic models with these covariates added to age, sex, and race/ethnicity (complete-case analyses among participants with non-missing expression for the gene under study; n = 102 for ACE2, n = 102 for TMPRSS2, n = 101 for FURIN, and n = 98 for NRP1). Continuous expression remained non-significantly associated with previous severe disease for all four genes (FURIN OR per 100 units = 1.38, 95% CI 0.88–2.16, *p* = 0.160; ACE2 OR = 1.01, 95% CI 0.97–1.06, *p* = 0.642; NRP1 OR = 1.00, 95% CI 0.98–1.02, *p* = 0.985; TMPRSS2 OR = 0.90, 95% CI 0.55–1.48, *p* = 0.686). In tertile sensitivity models, the intermediate ACE2 association was attenuated and no longer statistically significant (OR = 0.32, 95% CI 0.10–1.04, *p* = 0.058), whereas the intermediate NRP1 tertile remained associated with lower odds of previous severe disease (OR = 0.19, 95% CI 0.05–0.72, *p* = 0.014).

### 3.5. Exploratory Associations with Olfactory and Gustatory Dysfunction

As an exploratory assessment of post-acute neurosensory sequelae available in this cohort, we evaluated associations between post-acute receptor expression and self-reported olfactory dysfunction or dysgeusia, adjusting for age, sex, and race/ethnicity. No continuous-expression association reached statistical significance for either outcome (all *p* > 0.14). Tertile models likewise showed no statistically significant associations; intermediate NRP1 was not significantly associated with olfactory dysfunction (OR = 0.49, 95% CI 0.15–1.63, *p* = 0.244) or dysgeusia (OR = 0.47, 95% CI 0.14–1.57, *p* = 0.221).

## 4. Discussion

Given the cross-sectional design and post-infection timing, these results are associative and hypothesis-generating; they do not show that post-acute expression predicted or caused acute severity. The biological rationale for measuring expression after recovery is that convalescent upper-airway transcript levels may capture durable host epithelial phenotypes, either residual traits related to prior susceptibility or persistent injury/repair remodeling after severe illness, rather than the rapidly fluctuating inflammatory transcriptome of acute infection. Distinguishing these alternatives requires serial sampling, which was unavailable here.

For FURIN, both continuous and tertile-based estimates were statistically non-significant; the present data therefore do not support an association with COVID-19 severity. A mechanistic rationale remains biologically plausible because FURIN cleaves and activates viral protein precursors and facilitates SARS-CoV-2 entry. Chan et al. [12] demonstrated the importance of FURIN-mediated spike processing, while Li et al. and Zhang et al. [17,18] discussed FURIN expression in oncological, neurodegenerative, and neuropsychiatric conditions. Nevertheless, these mechanistic observations should not be used to infer an effect in this cohort. The non-significant estimates may reflect limited statistical power, unmeasured clinical or biological covariates, or the fact that expression was measured after recovery rather than during acute infection.

The inverse association observed for the intermediate ACE2 tertile was counterintuitive because ACE2 is both the principal viral entry receptor and an enzyme with protective physiological roles in the renin–angiotensin system [4,5,15,19]. Age-related differences and lower nasal ACE2 expression in children have also been reported [6,20], although findings vary across tissues and methods. One speculative explanation is a non-linear balance: very low ACE2 expression could weaken its normal vasoprotective and anti-inflammatory functions, intermediate expression could preserve those functions, and very high expression during acute infection could provide more binding sites for viral entry. However, the high ACE2 tertile was not significantly associated with severity, and post-acute measurements cannot test this proposed acute-phase mechanism. The pattern should therefore be considered hypothesis-generating rather than evidence of a protective threshold.

TMPRSS2 expression was not significantly associated with previous COVID-19 severity, despite its established role in spike-protein priming and viral entry. Kimura et al. and Lukassen et al. [7,8] described links between airway inflammation and ACE2/TMPRSS2 expression, while Sungnak et al. and Xu et al. [21,22] localized these entry factors to nasal, ciliated, goblet, and oral epithelial cells. A systematic review by Lechien et al. [23] likewise documented heterogeneous expression across head and neck tissues. The current findings do not negate that biological role; they indicate only that post-acute TMPRSS2 mRNA levels were not associated with previous severity in this cohort.

NRP1 represents another biologically relevant SARS-CoV-2 entry-related factor. Its role in viral entry has prompted interest in NRP1 as a potential therapeutic target, and computational screening studies have identified candidate compounds capable of interacting with NRP1 that may warrant further investigation as anti-SARS-CoV-2 agents [24]. Cantuti-Castelvetri et al. demonstrated that NRP1 can facilitate SARS-CoV-2 cell entry and infectivity, particularly through interaction with the FURIN-cleaved spike protein [25]. Neuropilin-1 is also a multifunctional cell-surface receptor involved in several signaling pathways and cellular processes [26]. In the present study, the inverse association observed for the intermediate NRP1 tertile provides some biological interest but should be interpreted cautiously. The absence of a corresponding significant association in the highest tertile argues against a simple dose–response relationship, and the post-acute timing of sampling prevents determination of whether the observed pattern reflects pre-existing susceptibility, a consequence of disease severity, or subsequent epithelial remodeling. Thus, the NRP1 finding should be regarded as exploratory and hypothesis-generating rather than evidence of a protective expression range.

Olfactory dysfunction was reported by 37.5% of this cohort; however, the study was not designed to establish a symptom-specific NRP1 mechanism, and routine swabs do not selectively sample the olfactory neuroepithelium. Consistent with that limitation, exploratory adjusted analyses found no statistically significant association between post-acute ACE2, TMPRSS2, FURIN, or NRP1 expression and olfactory dysfunction or dysgeusia. Therefore, although post-acute neurosensory sequelae are clinically important, the present receptor measurements do not appear to explain anosmia or dysgeusia in this cohort and do not support repositioning the primary analysis solely around long-COVID sensory outcomes.

An alternative interpretation is that the post-acute expression patterns reflect persistent epithelial or immune remodeling caused by the preceding illness rather than pre-existing susceptibility. Recent single-cell studies have demonstrated age-dependent injury and repair states in SARS-CoV-2-infected nasal epithelium and persistent aberrant differentiation, ciliated-cell loss, and inflammatory signaling in post-COVID nasal tissue [27,28]. Although those studies examined different populations and time points, they show that convalescent nasal expression need not represent the pre-infection baseline. Larger multicenter cohorts with serial pre-acute or early acute and convalescent sampling, healthy controls, and richer data on smoking, comorbidities, medication, vaccination, and viral variants are required to distinguish susceptibility from sequelae and to validate the present exploratory findings.

### Methodological Considerations

This study has several limitations that should be considered when interpreting the findings.

First, statistical power was constrained by the modest sample size, which was partly attributable to participant refusal and movement restrictions during the Spanish State of Alarm. Small or moderate associations may therefore have gone undetected, and the significant tertile estimates may be imprecise.

Second, because all participants were recruited from a single region (the Region of Murcia, southeastern Spain), the external validity of these results to other ethnic groups, geographic areas, or healthcare environments may be limited.

Third, gene-expression measurements were obtained only during post-acute convalescence, at a median of 75 days after symptom onset, once participants had recovered clinically. Although this timing reduced the immediate effects of active viral replication, acute inflammation, and transient mucosal injury, it precludes conclusions about expression during acute infection. Temporal direction cannot be established: the measured levels may represent pre-existing susceptibility, recovery-related normalization, or persistent remodeling caused by severe disease. Reverse causation therefore cannot be excluded.

Fourth, expression was categorized using sample-derived tertiles. This approach retained more participants per category than quartiles and allowed exploration of non-linear patterns, but the cutoffs remain data-dependent and potentially unstable. The tertile analyses were exploratory and involved multiple comparisons, increasing the possibility of chance findings.

Fifth, primary models adjusted for age, sex, and race/ethnicity. Smoking status, hypertension, diabetes mellitus, and Charlson comorbidity index were available and were examined in sensitivity analyses; however, medication use, detailed immunological status, obesity coding completeness, viral variant, and other clinical covariates remained incompletely captured. Residual and unmeasured confounding may therefore still have influenced both post-acute expression and previous disease severity. In sensitivity models, the intermediate ACE2 association was attenuated, underscoring the importance of comorbidity adjustment.

Sixth, individual SARS-CoV-2 variant and vaccination data were not systematically available. Recruitment occurred during the first two pandemic waves, when vaccination coverage was limited, and records were incomplete, but unrecorded vaccination before infection or sampling cannot be excluded. Variant- and vaccination-related differences could have affected mucosal expression and clinical severity.

Seventh, RNA quality metrics, including RNA Integrity Number values and A260/A280 ratios, were not systematically recorded during sample processing. Their absence limits assessment of RNA integrity and assay comparability. In addition, exact oligonucleotide primer sequences for ACE2, TMPRSS2, FURIN, NRP1, and GAPDH were not retained in the archived laboratory records available to the authors and therefore cannot be reported; amplification specificity was supported by melting-curve analysis and product verification as described in the Methods.

Eighth, swab-based sampling predominantly captures respiratory epithelial cells and does not selectively represent the olfactory neuroepithelium. Cellular composition was not independently quantified, so between-participant differences in sampled cell populations may have influenced normalized transcript levels.

Finally, the absence of an uninfected healthy control group, pre-infection baseline samples, and an external calibration standard prevented determination of whether expression had returned to baseline or remained persistently altered after infection. Expression could therefore be interpreted only as normalized relative units within this cohort, precluding direct fold-change comparisons with control-referenced studies.

Despite these limitations, this study also has several strengths.

The multicenter recruitment strategy strengthens the robustness of the findings and improves the representativeness of the study cohort compared to single-center designs.

Diagnostic certainty was ensured through the inclusion of only PCR-confirmed COVID-19 cases.

Furthermore, the direct molecular quantification of ACE2, TMPRSS2, FURIN, and NRP1 mRNA expression provides objective data on post-acute host-factor biology that may be relevant to prior COVID-19 severity.

The application of multivariate-adjusted statistical models adds analytical rigor and enhances the credibility of the reported associations.

Finally, this study contributes real-world evidence from a clinical cohort assembled during active pandemic conditions, offering novel insight into the nasal mucosal receptor expression landscape of individuals who have recovered from SARS-CoV-2 infection.

## 5. Conclusions

In this multicenter cross-sectional study, the primary adjusted analyses using continuous measures found no statistically significant association between post-acute upper-airway expression of ACE2, TMPRSS2, FURIN, or NRP1 and previous COVID-19 severity. Lower odds of severe disease were observed only for the intermediate ACE2 and NRP1 tertiles in exploratory analyses. Because samples were collected at a median of 75 days after symptom onset and no healthy or pre-infection comparator was available, these levels cannot be interpreted as predictors of acute severity or as evidence of a protective biological threshold. They may instead reflect persistent molecular remodeling after more severe disease, and reverse causation cannot be excluded. Larger longitudinal studies incorporating acute-phase and serial post-acute sampling, healthy controls, and comprehensive clinical covariates are needed to determine temporal direction and validate these findings.

## Figures and Tables

**Table 2:** Demographic, clinical, and post-acute gene-expression characteristics of the study participants.

Variables		Database with Missing Data	Imputed Database
Age	Median (IQR)	58.5 (16.2)	58.5 (16.2)
Sex	Male (%)	69 (66.3)	69 (66.3)
	Female (%)	35 (33.7)	35 (33.7)
Race/Ethnicity	Caucasian (%)	85 (81.7)	85 (81.7)
	Latino (%)	19 (18.3)	19 (18.3)
FURIN (relative units)	Median (IQR)	212.3 (105.1)	213.0 (103.8)
ACE2 (relative units)	Median (IQR)	2098.3 (1271.3)	2098.3 (1311.7)
NRP1 (relative units)	Median (IQR)	1904.3 (1627.6)	1897.7 (1599.8)
TMPRSS2 (relative units)	Median (IQR)	113.0 (54.4)	113.0 (54.0)
COVID-19 Severity (Status) ^†^	No hospitalization (%)	40 (38.5)	40 (38.5)
	Hospitalization with oxygen (%)	21 (20.2)	21 (20.2)
	Non-invasive ventilation or high-flow oxygen (%)	32 (30.8)	32 (30.8)
	Invasive ventilation (%)	11 (10.6)	11 (10.6)
COVID-19 severity (ventilatory support) ^†^	No ventilatory support (%)	61 (58.7)	61 (58.7)
	Ventilatory support (%)	43 (41.3)	43 (41.3)
COVID-19 severity (invasive ventilation) ^†^	No invasive ventilation (%)	93 (89.4)	93 (89.4)
	Invasive ventilation (%)	11 (10.6)	11 (10.6)
COVID-19 severity (hospital admission) ^†^	Hospitalization (%)	64 (61.5)	64 (61.5)
	No hospitalization (%)	40 (38.5)	40 (38.5)
Gustatory dysfunction (dysgeusia)	No (%)	66 (63.5)	66 (63.5)
	Yes (%)	38 (36.5)	38 (36.5)
Olfactory dysfunction	No (%)	63 (61.8)	65 (62.5)
	Yes (%)	39 (38.2)	39 (37.5)

^†^ According to the World Health Organization Clinical Progression Scale [16]. ACE2, angiotensin-converting enzyme 2; COVID-19, coronavirus disease 2019; FURIN, furin paired basic amino acid cleaving enzyme; IQR, interquartile range; NRP1, neuropilin-1; TMPRSS2, transmembrane protease-serine 2.

**Table 3:** Association between continuous post-acute gene expression and severe COVID-19 in binary logistic regression models.

	Imputed Database		
	COVID-19 Severity ^‡^		
Predictor	OR	95% CI	*p*
Unadjusted model			
FURIN (per 100 units)	1.34	0.90 to 2.00	0.152
Adjusted model ^†^			
FURIN (per 100 units)	1.38	0.88 to 2.17	0.157
Unadjusted model			
ACE2 (per 100 units)	1.02	0.98 to 1.06	0.401
Adjusted model ^†^			
ACE2 (per 100 units)	1.00	0.96 to 1.05	0.890
Unadjusted model			
NRP1 (per 100 units)	1.00	0.98 to 1.02	0.798
Adjusted model ^†^			
NRP1 (per 100 units)	1.00	0.98 to 1.02	0.788
Unadjusted model			
TMPRSS2 (per 100 units)	0.86	0.55 to 1.36	0.517
Adjusted model ^†^			
TMPRSS2 (per 100 units)	0.93	0.57 to 1.51	0.764

^†^ Adjusted for age, sex, and race/ethnicity. ^‡^ Severe COVID-19 was defined as high-flow oxygen, non-invasive ventilation, or invasive mechanical ventilation (WHO groups 4–5), versus no ventilatory support or low-flow oxygen (groups 1–3) [16]. ACE2, angiotensin-converting enzyme 2; CI, confidence interval; COVID-19, coronavirus disease 2019; FURIN, furin paired basic amino acid cleaving enzyme; NRP1, neuropilin-1; OR, odds ratio; *p*, *p* value; TMPRSS2, transmembrane protease-serine 2.

**Table 4:** Association between tertiles of post-acute gene expression and severe COVID-19 in binary logistic regression models.

	Imputed Database		
	COVID-19 Severity ^‡^		
Predictor	OR	95% CI	*p*
Unadjusted model			
Low FURIN	Ref.		
Intermediate FURIN	2.68	1.00 to 7.21	0.051
High FURIN	1.77	0.67 to 4.69	0.249
Adjusted model ^†^			
Low FURIN	Ref.		
Intermediate FURIN	1.70	0.55 to 5.25	0.356
High FURIN	1.90	0.65 to 5.54	0.237
Unadjusted model			
Low ACE2	Ref.		
Intermediate ACE2	0.58	0.21 to 1.58	0.285
High ACE2	1.33	0.52 to 3.40	0.547
Adjusted model ^†^			
Low ACE2	Ref.		
Intermediate ACE2	0.17	0.05 to 0.61	0.007
High ACE2	0.78	0.26 to 2.32	0.652
Unadjusted model			
Low NRP1	Ref.		
Intermediate NRP1	0.46	0.17 to 1.22	0.120
High NRP1	0.79	0.30 to 2.08	0.632
Adjusted model ^†^			
Low NRP1	Ref.		
Intermediate NRP1	0.29	0.10 to 0.88	0.030
High NRP1	0.58	0.20 to 1.72	0.331
Unadjusted model			
Low TMPRSS2	Ref.		
Intermediate TMPRSS2	1.05	0.40 to 2.74	0.921
High TMPRSS2	1.13	0.43 to 2.92	0.808
Adjusted model ^†^			
Low TMPRSS2	Ref.		
Intermediate TMPRSS2	1.17	0.41 to 3.30	0.771
High TMPRSS2	1.02	0.35 to 2.93	0.977

^†^ Adjusted for age, sex, and race/ethnicity. ^‡^ Severe COVID-19 was defined as high-flow oxygen, non-invasive ventilation, or invasive mechanical ventilation (WHO groups 4–5), versus no ventilatory support or low-flow oxygen (groups 1–3) [16]. ACE2, angiotensin-converting enzyme 2; CI, confidence interval; COVID-19, coronavirus disease 2019; FURIN, furin paired basic amino acid cleaving enzyme; NRP1, neuropilin-1; OR, odds ratio; *p*, *p* value; Ref., reference category; TMPRSS2, transmembrane protease-serine 2.

## Data Availability

The datasets used and/or analysed during the current study are available from the corresponding author on reasonable request.

## Abbreviations

The following abbreviations are used in this manuscript:

Abbreviation	Definition
ACE2	Angiotensin-converting enzyme 2
cDNA	Complementary DNA
CI	Confidence interval
COVID-19	Coronavirus disease 2019
FURIN	Furin paired basic amino acid cleaving enzyme
GAPDH	Glyceraldehyde-3-phosphate dehydrogenase
IQR	Interquartile range
MICE	Multivariate imputation by chained equations
NRP1	Neuropilin-1
OR	Odds ratio
PCR	Polymerase chain reaction
RNA	Ribonucleic acid
SARS-CoV-2	Severe acute respiratory syndrome coronavirus 2
TMPRSS2	Transmembrane protease-serine 2
WHO	World Health Organization
